# A novel apoptosis-inducing metabolite isolated from marine sponge symbiont *Monascus* sp. NMK7 attenuates cell proliferation, migration and ROS stress-mediated apoptosis in breast cancer cells†

**DOI:** 10.1039/c8ra09886g

**Published:** 2019-02-18

**Authors:** Sirpu Natesh Nagabhishek, Arumugam Madankumar

**Affiliations:** Cancer Biology Lab, Molecular and Nanomedicine Research Unit, Sathyabama Institute of Science and Technology Chennai-600119 Tamil Nadu India madankumarbio@gmail.com madankumar@sathyabama.ac.in +919942110146

## Abstract

The marine environment has a remarkable source of natural products mainly from marine fungi, which have been a central source of novel pharmacologically bioactive secondary metabolites. In this study, the search for a new potential apoptosis-inducing metabolite is focused on marine sponge-associated symbionts. A total of sixteen different sponges were obtained from the Gulf of Mannar region, India, and twenty-three different marine fungal strains were isolated and tested for antiproliferative activity by the MTT assay. Out of these, *Monascus* sp. NMK7 associated with the marine sponge *Clathria frondifera* was found to have a promising antiproliferative property. Furthermore, to isolate the pure active metabolite, the crude material was subjected to column chromatography and HPLC. Structural characterization was conducted by a variety of spectroscopic techniques including UV, IR, MS and NMR. The obtained results from the MS and NMR spectroscopy determined 418.5 Da to be the molecular weight and C_24_H_34_O_6_ to be the molecular formula of the metabolite, indicating the presence of monacolin X (NMKD7). NMKD7 was found to induce dose-dependent cytotoxicity in different human breast cancer cell lines MCF-7, T47D, MDA-MB-231, MDA-MB-468 and MCF-10A normal breast cell after 24 h of exposure. For elucidating the possible mode of cell death, T47D and MDA-MB-468 cells were treated with NMKD7 for 24 h to examine the morphological change of the chromatin (PI & AO/EB). Therefore, it has been suggested as the possible mechanism of apoptosis, and apart from this, it has also exhibited antibacterial and anti-migratory properties as well as induced the ROS stress (DCFH-DA), which causes the mitochondrial membrane potential difference (Rhodamine-123), the loss of cell membrane integrity and eventually cell death. Thus, the present study features a novel promising apoptosis-inducing metabolite (NMKD7) with minimal toxicity, suggesting its potential for biotechnological applications, and substantiates that it should be further considered for the elucidation of molecular targets and signal transduction pathways.

## Abstract 2

The marine environment has a remarkable source of natural products mainly from marine fungi, which have been a central source of novel pharmacologically bioactive secondary metabolites.

## Graphical abstract

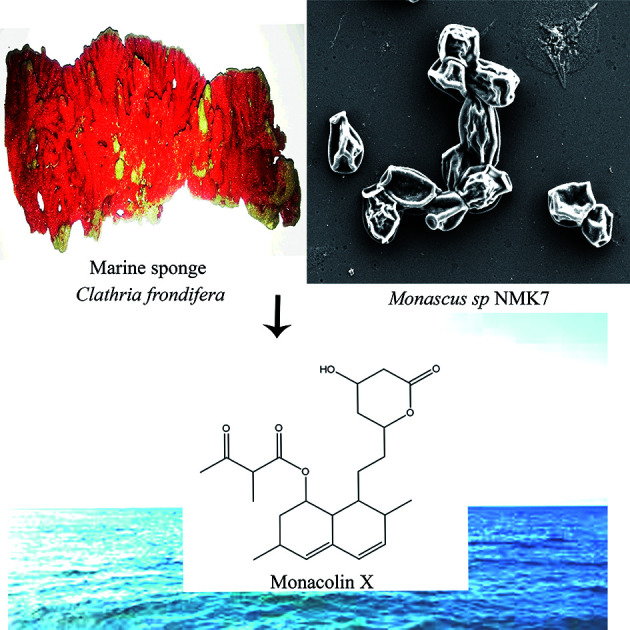

## Introduction

The increasing incidences of cancer have led to a global burden to find many diverse therapeutic treatments including surgery, radiation and chemotherapy.^1^ A large portion of the drugs are targeted for the signaling pathways responsible for cell proliferation and survival.^2^ Cancer fungotherapy is a promising scientific field that deals with antitumor metabolites derived from fungi,^3^ the initiation of apoptosis and the limiting of stifling cell proliferation in cancer cells with minimal or no side effects. Sponge-associated fungi metabolites have produced therapeutic compounds that have opened up a new era in marine pharmacology.^4^

Marine fungal metabolites are in the limelight of the drug discovery area as they are very effective therapeutic agents. There are numerous marine fungal metabolites discovered from a vivid source, and they demonstrate a wide range of potent biological activities including anticancer, antibacterial and antiviral.^5^ Marine fungi, compared to terrestrial fungi, are much less explored and have very good potential for drug discovery in addition to natural product discovery.^6,7^ Natural products are biologically active substances and are produced in relatively small amounts from rare species of animal or plants whose natural population cannot sustain the extensive collections needed for clinical trials.^8^ Natural products from the unique environments of oceans and seawater represent new microbes, which are unfamiliar and potent producers of secondary metabolites. Thus, the microorganisms for the screening of bioactive natural products can be considered as an alternative strategy, since it can be an effective approach.^9–12^ Marine sponges are the hosts for large population including actinomycetes, bacteria and fungi which comprise much as 40% of the sponge tissue's volume and helps in the stabilization of the sponge skeleton, allowing nutrient acquisition, the processing of metabolic waste and secondary metabolite production.^13^ Marine sponges are sessile soft-bodied organisms and they rely on chemical defenses through the production of secondary metabolites that are often from the associated microorganisms.^14^ Many sponge-associated fungi are distinguishable from their terrestrial analogues, compounds reported from *Aspergillus* or *Penicillium* marine fungi isolates are remarkably different from those of their terrestrial counterparts.^15^ This suggests the possibility of horizontal gene transfer through evolution. Marine fungi growing in a unique and stressful habitat develop the ability to produce structurally complex and unusual secondary metabolites due to their different transcriptome; proteasome; and finally, their different metabolome, which allows an organism to survive.^16^ Besides, some of the blockbuster drugs are of fungal origin such as β-lactam, the world's bestselling antibiotic, and cholesterol biosynthesis inhibitors. The anticancer drug paclitaxel was initially isolated from a plant source but was also found in endophytic fungi like *Taxomyces andreanae*^17^ and podophyllotoxin, an anticancer drug precursor.^18^ Hence, there is an increasing recognition for fungal secondary metabolites as a good resource for new drug leads, especially in the area of cancer research.^19^

Though many natural compounds are widely in use for chemotherapy, the application of such products in folk and traditional medicine has always been an important source of compounds with therapeutic potential^3^ and has opened the door to look towards several more new promising sources of marine natural products as a potential source of pharmaceuticals. Thus, in this study, we have isolated an apoptotic property-bearing compound produced by a marine sponge-associated fungi and have assessed its anticancer activity in breast cancer cells.

## Materials and methods

### Reagents and chemicals

All chemicals were purchased from Sigma-Aldrich (USA) and Himedia Laboratories Pvt. Ltd India, unless stated otherwise. Cell culture plastics were purchased from Tarsons Products (P) Ltd, India.

### Sample collection and sponge identification

Sixteen different marine sponges were collected (Sp1 to Sp16) (data not shown) as entangled specimens from a bottom trawl fish net in the Gulf of Mannar region at 6 AM (latitude-8°41′35.5′′N; longitude-78°08′21.7′′E). The water salinity was 36 ppt, and the temperature was 30.7 °C. The sponge sample was carefully removed from the wires of the trawl net and washed with seawater to remove any sand or adhered debris. Small pieces of samples were cut and transferred into a sterile sample container and transported to the laboratory under the required aseptic conditions, later kept in an icebox, correctly labelled and stored at 4 °C.^20^

A part of the sponge material was preserved in 70% methanol for sponge identification purposes. Based on the color, morphology and spicule pattern of the sponge, the identification was carried out.^21^

### Isolation of sponge-associated fungi and identification

The sponges were washed thoroughly with UV-treated sterile seawater until no visible debris was seen. 1 g of the central core of the sponge tissue was cut and homogenized with 99 mL of phosphate-buffered saline using mortar and pestle. The homogenate was serially diluted and plated on PDA, SDA and MA40S (Osmophilic Agar) to isolate the fungi, using a dilution series of 10^−5^ by a spread plate technique, and the plates were incubated at 28 °C for 10 days. From this method, 23 different fungi based upon morphology were isolated and checked for their antiproliferative activity. Only the strain showing potent antiproliferative activity was taken further for 18S rRNA sequencing,^22,23^ and by its morphological characteristics (*e.g.*, the shape of the conidia, colony color), was identified as *Monascus* sp. NMK7 (GenBank accession no. MG793201). The sequences obtained were aligned using ClustalW and phylogenetically analyzed using MEGA7 with a neighbor-joining tree.^24^ The fungi surface morphology was analysed using field emission scanning electron microscopy.

### Inoculum preparation, fermentation and extraction

The isolated culture plate of *Monascus* sp. NMK7 was taken, and its spores were inoculated into 250 mL Erlenmeyer flasks containing 100 mL of the MA40S (maltose 40 g L^−1^, sucrose 400 g L^−1^, and seawater to make up to 1 L) broth. After being cultivated for 72 h on a rotary shaker with 180 rpm at 28 °C, the MA40S (60 mL) was transferred to 54 × 2 L Erlenmeyer flasks each with 600 mL of liquid culture medium and were incubated at 28 °C with shaking at 180 rpm for 8 days. The fermented broth was filtered, and the filtrate was concentrated to one-twentieth of the original volume in a vacuum evaporator at a temperature below 50 °C (rotary vacuum evaporator), which resulted in a dark brown syrup. The crude material was extracted in ethyl acetate and concentrated to dryness in a rotary vacuum evaporator. Further, the soluble fraction was subjected to repeated silica gel column chromatography to purify the compound.

### Purification of the crude extract by column chromatography and RP-HPLC

The crude extract of *Monascus* sp. NMK7 was subjected to column chromatography using silica gel (mesh size: 60) using the solvent system hexane and methanol 0–100%. Then different elutions were collected in different test tubes, concentrated and assayed for antiproliferative/cytotoxic activity using MCF7 cell lines. The most active antiproliferative fraction (semi-purified) was taken to HPLC for further purification of the active compound. Further, only the active peak elusion purified cytotoxic fraction was subjected to the reverse-phase high-performance liquid chromatography (RP-HPLC) system using the C_18_ column (Sunfire-C18, 4.6 × 250 mm), with the photodiode array (PDA) detector (Waters). The mobile phase was applied as the linear gradient of the following: detection – PDA (200–800 nm) with the detection wavelength of 238 nm; mobile phase: A – water, B – acetonitrile; (0–2 min) 70% A : 30% B, (2–10 min) 10% A : 90% B, (10–20 min) 50% A : 50% B, (20–30 min) 70% A : 30% B with a flow rate of 1 mL min^−1^.

### Structural characterization

#### MS analysis

Mass spectrometer equipped with electrospray ionization with both positive and negative modes was scanned in the mass range of *m*/*z* = 200–2000. The mass of the HPLC purified compound was determined by LC-MS/MS operated in positive mode electrospray ionization (ESI) with a resolution of 22 500 FWHM. The capillary and cone voltage were set to 3 kV, and the source temperature and desolvation temperature were set to 140 and 400 °C, respectively. The gas flow of the cone was 50 L h^−1^, and a desolvation gas flow of 1000 L h^−1^ was set to analyze the mass of the HPLC-purified compound. The ESI data were processed by a Thermo Qual browser, and the collision energy was applied for fragmentation.

#### NMR analysis

^1^H and ^13^C NMR measurements were performed on a Bruker Avance 300 MHz instrument at 25 °C in deuterated chloroform, and the chemical shift and coupling constant values were reported on the *δ* (ppm) & *J* (Hz) scale relative to TMS. Based on the obtained results, the purified compound was termed as NMKD7.

#### FT-IR analysis

FT-IR analysis is essential for the identification of the functional groups present in a compound. The pure compound was characterized using IR-grade KBr (Thermo Nicolet Nexus 670 spectrometer), and the spectra were scanned over a range of 4000–500 cm^−1^ and recorded in the diffuse reflectance mode at a resolution of 4 cm^−1^.

#### Cell line and culture

MCF-7, T47D, MDA-MB-231 and MDA-MB-468 breast cancer cell lines and normal MCF-10A breast cell lines were obtained from NCCS, Pune, India. The cells were grown in T25 culture flasks containing DMEM and L-15 supplemented with 10% FBS and 1% antibiotics (100 U mL^−1^ penicillin and 100 μg mL^−1^ streptomycin). The cells were maintained at 37 °C in a humidified atmosphere containing 5% CO_2_. Upon reaching confluency, the cells were trypsinized and passaged to be used for further assay.

#### Screening of fungal extracts for anti-cancer activity

The antiproliferative effect of NMKD7 at various concentrations was studied on human breast cancer cell lines MCF-7, T47D, MDA-MB-231, MDA-MB-468 and normal MCF-10A cells and were compared to standard control doxorubicin (Dox) at 1.5 μM. The cells were grown in T25 culture flasks containing DMEM and L-15 supplemented with 10% FBS and 1% antibiotics (100 U mL^−1^ penicillin and 100 μg mL^−1^ streptomycin). The cells were maintained at 37 °C in a humidified atmosphere containing 5% CO_2_. Upon reaching confluence, the cells were detached using Trypsin–EDTA solution and were subcultured at a density of 5000 cells per well. At 50% confluence, the culture medium was aspirated, and the cells were treated with different concentrations (0, 10, 20, 40, 80, 150 and 250 μM) of NMKD7 for 24 h at 37 °C in the CO_2_ incubator. Later cells were incubated with XTT (1 mg mL^−1^) (2,3-bis[2-methoxy-4-nitro-5-sulfophenyl]-2*H*-tetrazolium-5-carboxyanilide inner salt) for 3 hours. The absorbance was measured at 450 nm with a standard microplate reader (EnSpire Perkin Elmer USA).
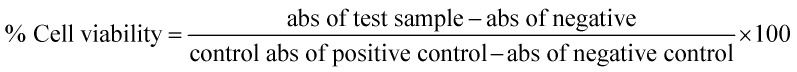


#### Cellular integrity measurement by LDH assay

Cell membrane integrity of the MCF-7, MDA-MB-231 and MCF-10A cell lines were evaluated by determining the activity of lactate dehydrogenase (LDH) leaking out of the cell according to the manufacturer's instructions (Pierce Thermo Scientific USA). The cytotoxicity was assessed quantitatively by measuring the activity of LDH in the supernatant. Briefly, the cells were exposed to different concentrations of NMKD7 for 24 h; then, 100 μL per well of each cell-free supernatant was transferred in triplicate into wells in a 96-well plate, and 100 μL of LDH assay reaction mixture was added to each well. After 3 h of incubation under standard conditions, the optical density of the colour generated was determined at a wavelength of 490 nm using a multimode plate reader (EnSpire Perkin Elmer USA).

#### Apoptotic studies

Cell and nuclear morphologies were evaluated using PI and AO/EB staining.^25^ The nuclear morphology was analyzed using bright field microscopy in T47D and MDA-MB-468 cells after treatment with NMKD7 at its IC_50_ concentration and Dox (1.5 μM) for 24 h, respectively. Control cells were grown in the same manner without NMKD7. The cells were trypsinized and fixed with ethanol; then, the cell nuclei were stained using 1 mg mL^−1^ propidium iodide (PI) at 37 °C for 15 min in darkness. Further characteristic apoptotic changes were determined by AO/EB staining.^26^ Coverslips were taken, kept on glass slides and stained with 100 μL of the dye mixture (1 : 1 ratio of AO and EB), and it was immediately viewed under an inverted fluorescence microscope (EVOS FL digital inverted fluorescence microscope (AMG)).

#### DCFH-DA staining

Intracellular ROS levels were measured using a cell-permeable fluorescent probe DCFH-DA (2′,7′-dichlorofluorescein diacetate). DCFH-DA diffuses through the cell membrane and is hydrolyzed by an intracellular esterase to the non-fluorescent dichlorofluorescein (DCFH), which is rapidly oxidized by ROS to fluorescent dichlorofluorescein. T47D and MDA-MB-468 cells (8 × 10^4^ cells per well in 24-well plates) were seeded in DMEM medium and incubated for 48 h at 37 °C with 5% CO_2_ to allow the cells to become semi-confluent. Semi-confluent T47D and MDA-MB-468 cells were treated with NMKD7 at the IC_50_ concentration and Dox (1.5 μM) as a standard control for 24 h. After the treatment period, DCFH-DA (in absolute DMSO) was added to the treated plates at a final concentration of 10 μM and incubated in the dark at 37 °C for 30 min. Post-staining, the plates were rinsed twice with PBS, and images were taken on an inverted fluorescent microscope (EVOS FL digital inverted fluorescence microscope (AMG)).^27^

#### Determination of mitochondrial membrane potential (MMP; Δ*ψ*_m_)

To determine the effect of NMKD7 on the electrical potential across the inner mitochondrial membrane on T47D and MDA-MB-468, Rhodamine 123 (R-123), a lipophilic, cationic indicator, was used. T47D and MDA-MB-468 (2 × 10^4^ cells per well in 24-well plates) were seeded in DMEM medium and incubated for 48 hours at 37 °C with 5% CO_2_ to allow the cells to become confluent. After this period, the cells were pre/co-administered with NMKD7 at the IC_50_ concentration and Dox (1.5 μM), respectively, for 24 h. The cells were then rinsed with PBS, and fresh media containing R-123 solution (10 μg mL^−1^) was added to the treated wells; the plates were then incubated in the dark at 37 °C for 20–30 min.^28^ Subsequently, the cells were washed twice with PBS, and the cell images were taken using a fluorescent microscope (EVOS FL digital inverted fluorescence microscope (AMG)).

#### Migratory assays

Cell migration was analyzed by a cell migration assay. The MDA-MB-468 and T47D cells were grown to confluence, and then a scratch was made in the monolayer by dragging a 200 μL pipette tip across the layer washed with PBS, which was then incubated in culture medium supplemented with 5% FBS. The cells were treated with NMKD7 at the IC_50_ concentration and Dox (1.5 μM). Migration into the open scar was documented with microphotographs at different time points after wounding. The number of migrating cells was quantified by counting all the cells within a 0.4 mm region in the center of each scratch. A minimum of 3 individual cultures were used to calculate the mean migratory capacity of each cell culture condition.^29^

#### Antibacterial assay

Minimum inhibitory concentrations (MIC) of extracts for antibacterial activity were determined using a broth microdilution assay as described in previous reports.^30^ Overnight cultures (incubated at 37 °C in an orbital shaker) of two Gram-positive (*Staphylococcus aureus* ATCC 25923 and *Enterococcus faecalis* ATCC 29212) and two Gram-negative (*Klebsiella pneumoniae* ATCC 13883 and *Pseudomonas aeruginosa* ATCC 15442) bacterial strains were diluted with sterile Mueller–Hinton (MH) broth to give final inoculums of approximately 10^5^ CFU mL^−1^. The dried compound from NMKD7 was suspended in ethanol to a stock concentration of 1 mg mL^−1^, and 1, 2, 4, 16, 32, 64 and 128 μg mL^−1^ of NMKD7 was added into a 96-well microtitre plate containing 200 μL per well of four bacterial strains and incubated at 37 °C for 24 h,further dilutions were made to find the exact MIC values. The bacterial growth was monitored by adding 50 μL of 0.2 mg mL^−1^*p*-iodonitrotetrazolium chloride (INT) (Sigma-Aldrich, Germany) and underwent further incubation at 37 °C for 24 h. Since the colourless tetrazolium salt was biologically reduced to a red product due to the presence of active organisms, the MIC values will be determined as the concentrations in the last wells in which no colour change was observed after adding the INT indicator. Minimum inhibitory concentrations (MICs) were determined in accordance with the Clinical and Laboratory Standards Institute (CLSI). Gentamicin (1–10 μg mL^−1^) dissolved in deionized water was used as an antimicrobial standard control.

#### Statistical analysis of biological assays

All results were presented as means ± standard deviation from triplicate experiments performed in a parallel manner. The entire group data were statistically evaluated using the SPSS software package 16. The statistical differences were determined using the Ducan's multiple range test. All comparisons were made relative to untreated controls. A statistically significant difference was considered at *P* < 0.05.

## Results

### Identification of NMKD7-producing fungi associated with the marine sponge

Initially, sixteen marine sponges (Sp1 to Sp16) were collected, and twenty-three associated fungi (NMK1-23) were isolated based on the colony morphology; each crude was then checked for anticancer activity on the MCF-7 breast cancer cell line (ESI Fig. 1A†). Based on the results, the NMK7 strain associated with the marine sponge (Sp4), showing the highest anticancer activity, was taken for identification.

**Fig. 1:**
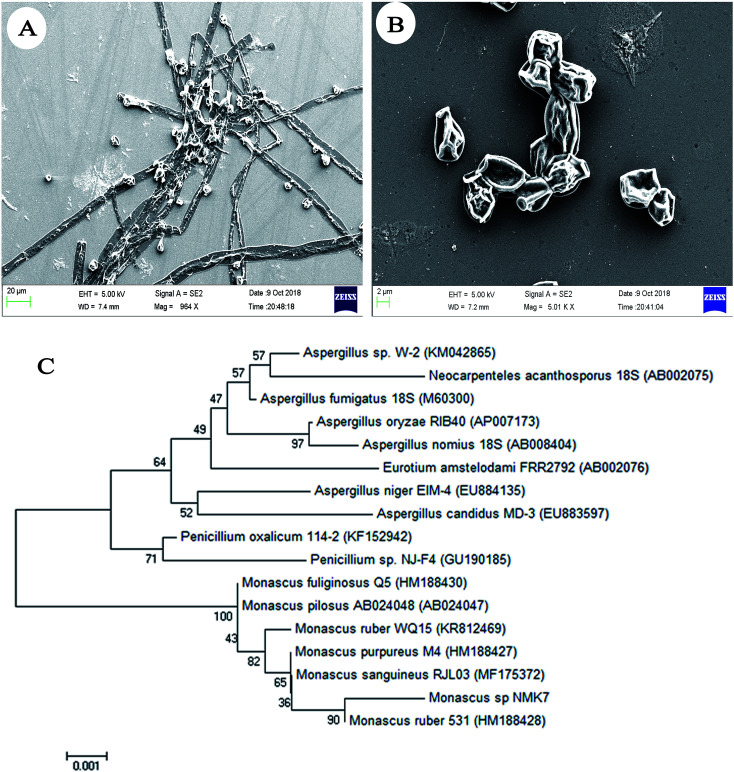
Scanning electron microscopic (SEM) images, (A) and (B) showing the surface morphology of fungi *Monascus* sp. NMK7 with a broadly ellipsoid body and a hyaline and smooth surface (15 000×). (C) Phylogenetic analysis of *Monascus* sp. NMK7 from the marine sponge *Clathria frondifera*. Maximum likelihood phylogenetic representation of NMK17 with the neighbor strains by MEGA7 software.

The marine sponge (Sp4) was taxonomically identified based on its morphology and the spicule structure as *Clathria frondifera* by Dr Siva Leela, a scientist in Zoological Survey of India (ZSI), MBRC, Chennai, Tamil Nadu, India. The fungal strain NMK7 was isolated from the marine sponge *Clathria frondifera* collected from Gulf of Mannar and was identified using 18S rRNA gene sequencing. The cell surface morphological analysis of the isolate was studied using scanning electron microscopy, which, in support, has shown that the matured, broadly ellipsoid, hyaline, smooth, and 5 μm size (Fig. 1A and B) for the maximum-likelihood phylogenetic analysis revealed that the strain belonged to clade *Monascus ruber* sp. and was assigned as the *Monascus* sp. NMK7 strain (Fig. 1C) and submitted into the GenBank with the accession number MG793201.

### Structural characterization

#### Initial purification by column chromatography and HPLC

**Fig. 2:**
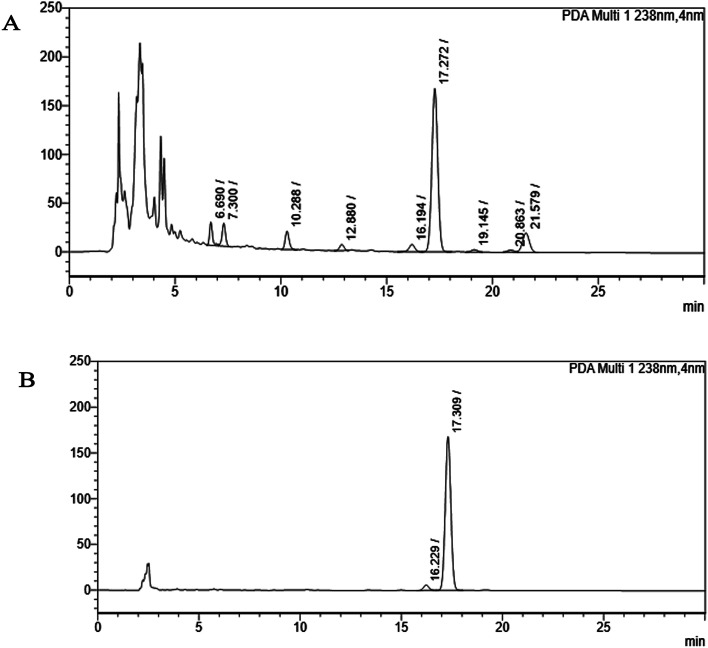
HPLC chromatogram (A) shows the partially purified crude extract of *Monascus* sp. NMK7 and represents 9 different peaks at 238 nm, indicating various other compounds present apart from the compound of interest. (B) HPLC chromatogram shows a single peak, indicating a pure isolated compound at 17.30 RRT at 238 nm.

The apoptosis-inducing effect of the crude ethyl acetate extract of *Monascus* sp. NMK7 was initially assessed for its cytotoxic activity in the MCF-7 cell line. The crude extract was subjected to silica gel column chromatography and fractionated into 12 tubes (F1 to F12), out of which fraction 4 (F4) had shown cytotoxicity towards the MCF-7 cell line (ESI Fig. 1B†). The active fraction 4 was analyzed by RP-HPLC, and the chromatogram revealed 9 different compounds (Fig. 2A). Each of the fractions (E1 to E9) were assessed for its cytotoxic activity in the MCF-7 cell line (ESI Fig. 1C†); 17.27 RRT (active peak (E6)) has shown the apoptosis-inducing effect at 238 nm. Further, the active peak was collected in large volumes and re-injected and tested for further impurities; the compound shown (Fig. 2B) was revealed to be a pure compound with a single peak. Further, the 17.27 RRT of several elutions was concentrated under high vacuum, dried and recrystallized in solvent (chloroform); the remaining aqueous part was subjected to a lyophilizer to obtain a pure compound, giving a white powdery product. The isolated solid compound NMKD7 was subjected to spectral analysis.

#### MS analysis

**Fig. 3:**
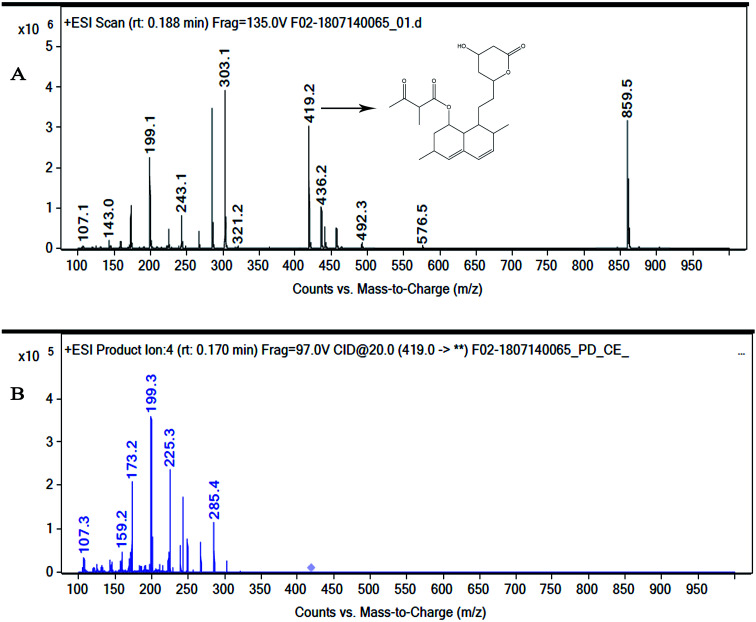
(A) Mass spectrum of NMKD7 (Monacolin X). (MS-ESI) showing a *m*/*z* = 419.2 [M + H] and 859.5 [2M + Na^+^]. The molecular mass was determined to be 418.5 Da. (B) MS fragmentation spectra of the monacolin X peak at *m*/*z* = 419.2 at a collision energy of 20, the structure for the peaks at 199.3, 173.2 and 159.2 represents the possible fragmentation form monacolin X.

The mass spectrum of the compound at 17.309 RRT in the positive ionization mode (Fig. 3A) exhibited an M + H^+^ peak at *m*/*z* = 419.2 [M + H^+^] and 859.5 [2M + Na^+^] atomic mass unit (amu), and the other major fragment ions are at *m*/*z* = 303.20, 285.1, 244.3, 192.3, 176.3, corresponding to the exact calculated molar mass of monacolin X (418.5).^31,32^ Thus, the exact mass of the active compound was identified as 418.2 Da.

#### NMR

##### ^1^H NMR spectroscopy

The isolated compound was dissolved in CDCl_3_ and characterized by ^1^H and ^13^C NMR spectroscopy. The ^1^H-NMR spectrum of the isolated impurity suggested the presence of the –COCH_3_ group and one –COCH(CH_3_)CO– group. Thus, the presence of these groups can be confirmed by locating a singlet of three protons at 2.25 ppm and a multiplet of one proton at 3.50–3.57 ppm. The isolated structure has three alkenes and a proton, which were confirmed by the ^1^H NMR spectrum. The corresponding [5-alkene–CH] proton corresponded to an observed doublet at 5.97 ppm, and the coupling constant value *J* = 9.9 Hz. The [4-alkene–CH] group proton was an observed quartet at 5.74–5.79 ppm, with a corresponding coupling constant value of 6.0 Hz, *J* = 6.3 Hz. The [6-alkene–CH] group proton was an observed triplet at 5.49 ppm, with a corresponding coupling constant value of *J* = 3.0 Hz (ESI Fig. 2A†).

^1^H-NMR spectrum (CDCl_3_, ppm) 5.97 (d, 1H, *J* = 9.9 Hz, 5-alkene–CH), 5.74–5.79 (q, 1H, *J* = 6.0 Hz, *J* = 6.3 Hz, 4-alkene–CH), 5.49 (t, 1H, *J* = 3.0 Hz, 6-alkene–CH), 5.35 (d, 1H, *J* = 3.0 Hz, 1-CH), 4.59–4.65 (m, 1H, 5′-CH), 4.32–4.37 (m, 1H, 3′-CH), 3.50–3.57 (m, 1H, 2′′-CH), 2.33–2.80 (m, 4H, –CH_2_&–CH), 2.25 (s, 3H, 4′′-CH_3_), 2.03–2.17 (m, 7H, –CH_2_&–CH), 1.80–1.99 (m, 4H, –CH_2_&–CH), 0.86–1.34 (m, 9H, 10,9,5′′-CH_3_).

##### ^13^C-NMR spectroscopy

The ^13^C-NMR spectrum of the isolated compound confirmed the carbonyl signal C-3′′ at 204.88 ppm and the corresponding methyl signal C-2′′ at 53.39 ppm. The 4′′-CH_3_ group carbon was observed at 27.33 ppm. The 1′′&1′-CO carbon was observed at 170.36 and 169.93 ppm. The [5-alkene–CH] carbon was observed at 129.27 ppm, and the corresponding [4-alkene–CH] group carbon was observed at 128.09 ppm. The [6-alkene–CH] group carbon was observed at 131.59 ppm (ESI Fig. 2B†).

^13^C-NMR spectrum (CDCl_3_, ppm): 204.88 (3′′-CO), 170.36 (1′′-CO), 169.93 (1′-CO), 133.34 (-CH), 131.59 (6-alkene–CH), 129.27 (5-alkene–CH), 128.09 (4-alkene–CH), 69.45 (–CH), 62.69 (–CH). 53.39 (2′′-CH), 38.60 (–CH_2_), 37.34 (–CH), 36.54 (–CH), 36.11 (–CH_2_), 32.59 (–CH_2_), 32.50 (–CH_2_), 30.91 (–CH), 30.60 (–CH), 29.52 (–CH), 24.15 (–CH_2_), 22.81 (–CH_3_), 13.78 (–CH_3_), 12.72 (–CH_3_).

**Fig. 4:**
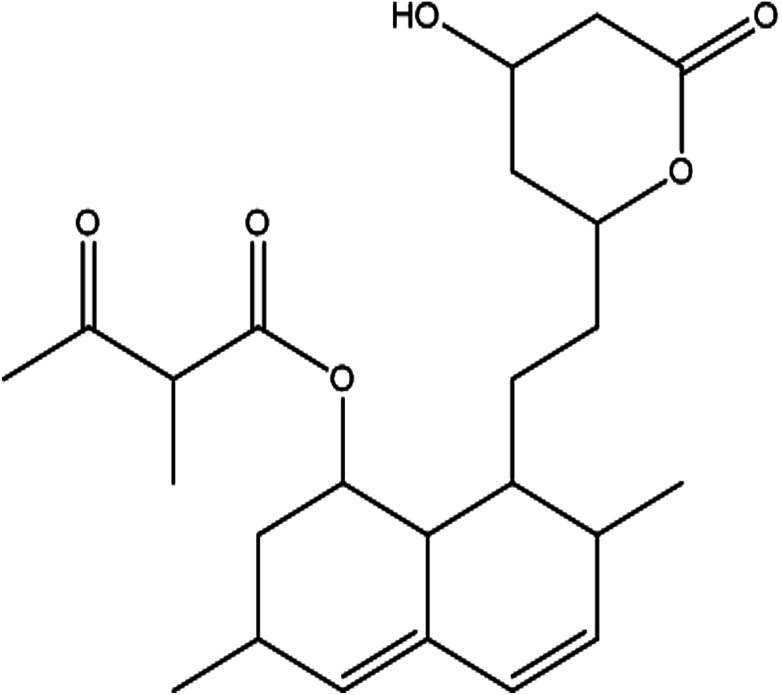
Structure of the isolated molecule monacolin X.

From the DEPT-135-^13^C NMR spectrum, the isolated product has five –CH_2_ carbons in its structure. The five –CH_2_ carbons were confirmed by the DEPT-135-^13^C NMR spectrum, and the five –CH_2_ corresponding values were 24.15 (7′-CH_2_), 32.49 (6′-CH_2_), 32.57 (4′-CH_2_), 36.09 (2-CH_2_), 38.59 (2′-CH_2_) (ESI Fig. 3†). The ^1^H-NMR, ^13^C and DEPT-135-^13^C NMR spectra revealed the molecular formula to be C_24_H_34_O_6_ and predicted the structure to be monacolin X (Fig. 4).^31^

#### FTIR

The FT-IR spectra of the pure compound presented characteristic peaks at 3419 cm^−1^ and 3703 cm^−1^ (O–H stretch vibration), 2927 cm^−1^ (C–H stretch vibration) and 1259 cm^−1^, 1063 cm^−1^ and 1719 cm^−1^ (stretch vibration of –C–O and –C=O carbonyl functional group), and 1612 cm^−1^ (C=C) (ESI Fig. 4A†). Thus, all these functional groups well matched the predicted structure of monacolin X.

#### UV-spectroscopy

The UV spectra of the impurity at 17.309 RRT were measured on a Jasco instrument in methanol, and the maxima were observed at 238.2 nm (ESI Fig. 4B†).

#### Cell viability assay

**Fig. 5:**
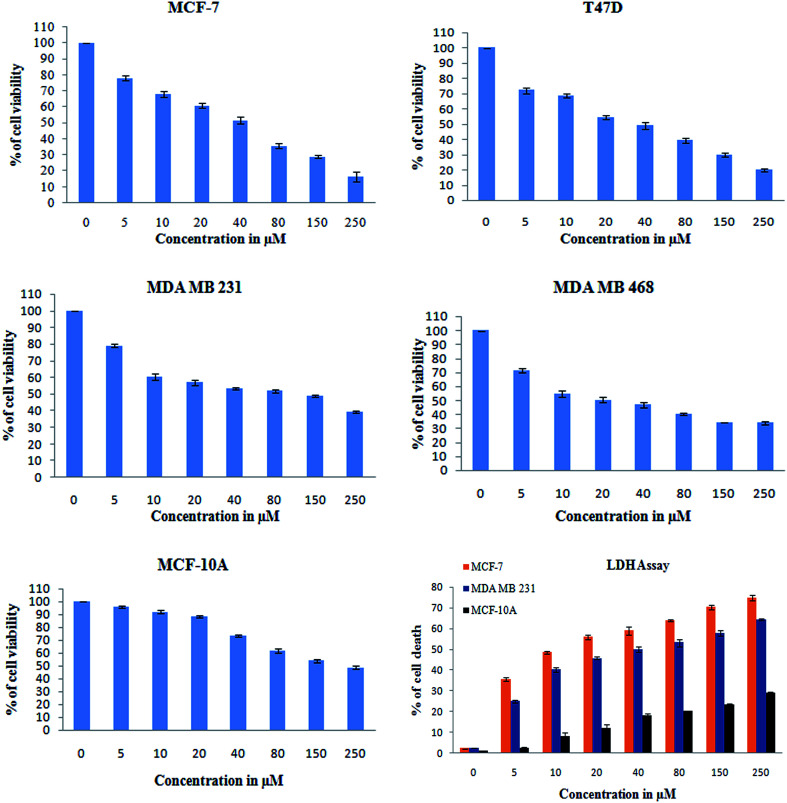
XTT assay was used to check for the antiproliferative and cytotoxic nature of monacolin X on human breast cancer cell lines MCF-7, T47D, MDA-MB-231, MDA-MB-468 and MCF-10A, a non-cancerous breast cell line. The cell viability was measured by XTT assay. All the cells were treated with monacolin X at various concentrations (0–250 μg mL^−1^) for 24 h. Cell membrane integrity by the release of lactate dehydrogenase (LDH) activity by LDH assay. MCF-7, MDA-MB-231 and MCF-10A cells were treated with monacolin X at various concentrations (0–250 μg mL^−1^) for 24 h. LDH released into the medium was measured along with blank, untreated cells (0 μM), showing low LDH release in media, whereas treated cells had a dose-dependent release of LDH.

To determine the effect of monacolin X (NMKD7) on the cell viability of breast cancer cells *in vitro*, ER-α-positive MCF-7 and T47D, ER-α-negative MDA-MB-231 and MDA-MB-468 cells were treated with increasing concentrations of monacolin X (0–250 μM for 24 h). Results have shown dose-dependent cytotoxicity on all the cell lines that have exhibited maximum sensitivity, showing a reduction in cell viability even at the lowest concentration (10 μM), indicating that the treatments were cytotoxic, which was comparable to that of the positive control Dox (1.5 μM). Treatment with monacolin X resulted in IC_50_ values for the MCF-7, T47D, MDA-MB-231, MDA-MB-468 and normal MCF-10A cell lines (42.87 ± 0.75, 33.90 ± 0.66, 48.78 ± 0.89, 26.34 ± 1.02 and 170.66 ± 0.89 μM, respectively (Fig. 5)). The cell viability was also measured by lactate dehydrogenase (LDH) release. As dead cells lose their membrane integrity, the enzymes present inside the cell leaks out, and their activity was measured externally (Fig. 5), indicating the leakage of LDH in the culture media after treatment with different concentrations of monacolin X. MCF-7 exposure to monacolin X raised the LDH leakage after 24 h treatment. The treatment regimens on breast cancer cell lines resulted in toxicity, in comparison with normal MCF-10A cell line with an IC_50_ concentration of 170 μM, demonstrating that monacolin X shows minimal toxicity on normal breast cell lines. Treatment with monacolin X has shown irregular and shrunken cell morphology along with significant reduction in cell number in a dose-dependent manner. This was observed using bright field phase contrast microscopy under 20× magnification.^33^

#### Morphological evidence of apoptosis by PI & AO/EB dual staining

Propidium iodide (PI) staining was used to show the morphological changes under a fluorescence microscope. The percentage of apoptotic nuclei after treatment with an IC_50_ concentration of monacolin X significantly increased when compared to the control (untreated cells) in T47D and MDA-MB-468 cells, which was comparable to that of the standard drug control (Dox) effect. This resulted in altered morphology such as nuclear fragmentation and chromatin condensation. The cells were scored at random and classified into apoptotic and non-apoptotic cells based on their nuclear morphology.

**Fig. 6:**
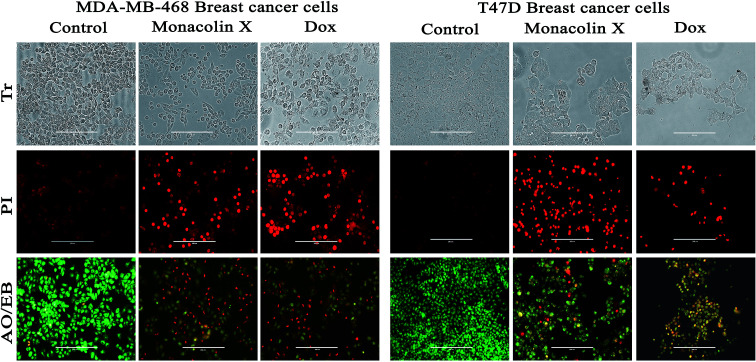
The morphological analysis of MDA-MB-468 and T47D breast cancer cell lines upon treatment with monacolin X (IC_50_) and Dox (1.5 μM) for 24 h. The morphological changes of treatment group of breast cancer cells showed irregular and shrunken cell morphology, whereas the control group showed normal cell morphology. PI staining by fluorescence microscopy. The percentage of necrotic nuclei after 24 hours of treatment increased enormously when compared to the control, as revealed by nuclear condensation and fragmentation. Apoptotic and nuclear morphological changes in MDA-MB-468 and T47D cells treated with monacolin X and Dox were evaluated with AO/EB dual staining. The stained cells were characterized to viable (light green), early apoptotic (yellow fluorescence and condensed chromatin), late apoptotic (orange fluorescence) and nonviable cells (red-colored fluorescence). In the control, there were only viable cells when compared to the treatment groups which had high numbers of apoptotic cells. Magnification (20×); the experiments were performed in triplicate.

Further, T47D and MDA-MB-468 cells, treated with an IC_50_ concentration of monacolin X and Dox (1.5 μM), showed significantly increased levels of apoptotic cells in comparison to the untreated cells, where no apoptotic cells were observed. This is evidenced by the acridine orange/ethidium bromide (AO/EB) differential staining method (Fig. 6). The stained cells were characterized to be viable (light green), early apoptotic (yellow fluorescence and condensed chromatin), late apoptotic (orange fluorescence) and nonviable cells (red colored fluorescence).^34^ Early apoptotic cells with nuclear margination and chromatin condensation, indicated in yellow colour, and late apoptotic cells with fragmented chromatin, indicated in orange colour, were noticed in monacolin X and Dox treated cells. While the control cells have shown intact nuclear architecture, the monacolin X treated cells have shown membrane blebbing, condensed nuclei, and apoptotic bodies.

#### Monacolin X modulates ROS production in T47D and MDA-MB-468 cells

**Fig. 7:**
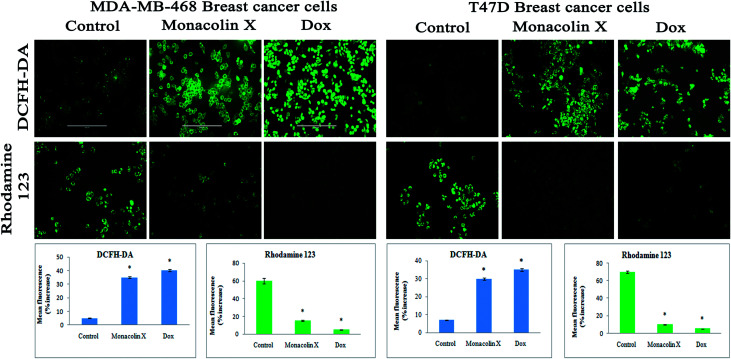
DCFH-DA and Rhodamine-123 staining for ROS and Δ*ψ*_m_. The images showed significant ROS generation upon treatment with monacolin X (IC_50_) and Dox (1.5 μM) treated on MDA-MB-468 and T47D breast cancer cell lines over 24 h. There was a significant difference in the control and treated groups, with high ROS generation in the treated group. Rhodamine-123 staining showed the mitochondrial membrane potential Δ*ψ*_m_ as decrease fluorescence was observed in monacolin X and Dox, indicating cell death. Magnification (20×); the experiments were performed in triplicate, and the data are expressed as mean ± SD; *p < 0.05, as compared to the control group, was considered as significant.

T47D and MDA-MB-468 cells treated with an IC_50_ concentration of monacolin X and Dox (1.5 μM) for 24 h have shown significantly increased levels of ROS in the cells as there was no ROS production in the control cells. This is evidenced by the DCFH-DA staining method (Fig. 7).

#### Rhodamine-123 stain

Fig. 7 shows the variations in mitochondrial membrane potential in the T47D and MDA-MB-468 cells, treated with an IC_50_ concentration of monacolin X and Dox (1.5 μM) for 24 h, which showed a significant decrease in MMP when compared to the control. This indicated a reduction in R-123 fluorescence in the case of treatment, whereas increased fluorescence was observed in the control, indicating that the treatment group was losing its mitochondrial membrane integrity. Thus, monacolin X induces reactive oxygen species (ROS) that have been implicated in the cellular response to stress and are involved in the mediation of apoptosis *via* mitochondrial DNA damage in breast cancer cell lines.^35^

#### Migrations of breast cancer cells

**Fig. 8:**
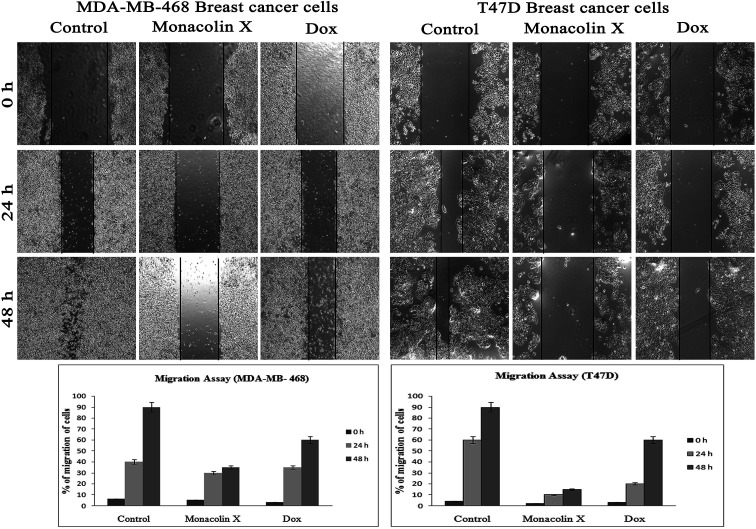
The anti-migratory property of monacolin X (IC_50_) and Dox (1.5 μM) treated on MDA-MB-468 and T47D breast cancer cell lines for 24 and 48 h. Significant differences between the control groups and treated groups in terms of the number of migrated cells were noted at 24 and 48 h. Treatment I showed fewer migrated cells compared to the control cancer cells. Magnification (4×). Experiments were performed in triplicate and the data were expressed as a mean ± SD; *p < 0.05, as compared to the control group, which was considered as significant.

To ascertain the inhibitory effect of monacolin X on breast cancer metastasis, we used T47D (low metastatic) and MDA-MB-468 (high metastatic) on migration through scratch assay. There was a reduced migration upon treatment with monacolin X and Dox (1.5 μM) when compared to the control untreated cells (Fig. 8), indicating monacolin X to have a very good anti-migratory property even on high metastatic cell MDA-MB-468. The assay was carried out for 48 h, and microscopic observation was taken at 0 h, 24 h and 48 h.

#### Antibacterial assay

*In vitro* antibacterial minimal inhibitory concentration of the monacolin X has shown a MIC of 38 μg mL^−1^ against the Gram-positive *Enterococcus faecalis* ATCC 29212 and a *Staphylococcus aureus* ATCC 25923 MIC of 20 μg mL^−1^. Monacolin X exhibited a MIC of the two Gram-negative pathogens of 18 μg mL^−1^*Pseudomonas aeruginosa* ATCC 15442 and *Klebsiella pneumoniae* ATCC 13883 of 120 μg mL^−1^ in comparison with standard Gentamicin (1–10 μg mL^−1^) (Table 1).

**Table 1:** Antimicrobial activity of monacolin X (MIC value expressed in μg mL^−1^)

Microorganism	MIC (μg mL^−1^)	
	Monacolin X	Gentamycin
*Enterococcus faecalis* ATCC 29212	38	3
*Staphylococcus aureus* ATCC 25923	20	2
*Pseudomonas aeruginosa* ATCC 15442	18	6
*Klebsiella pneumoniae* ATCC 13883	120	3

## Discussion

Marine-derived fungi synthesize enormous secondary metabolites with complex and unique structures.^36^ However, very little is known about the biological activity of the extracts and compounds of these fungi. The present work, explored the fungi *Monascus* sp. NMK7 associated with sponge *Clathria frondifera*, which secretes the anticancer/antiproliferative compound and polyketide monacolin X. This association of fungi to the host sponge could improve the chemical defense capability. *Monascus* sp. secretes many secondary metabolites like monascin; ankaflavin; monacolins K, J and L apart from a few other pigments; and polyketides.^37^ These bioactive compounds have demonstrated their high potential for the development of potent pharmaceutical products. This is the first report on *Monascus* sp. isolated from the marine sponge in contrast to the previous reports on the terrestrial isolates. Endo *et al.* reported that *Monascus ruber* M82121 a mutant strain treated with UV irradiation and *N*-methyl-*N*′-nitro-*N*-nitrosoguanidine, producing monacolin X along with monacolins J and L.^31^ In this present study, the isolated strain was capable of producing monacolin X without any other treatments or induced mutation. Monacolin X could be produced because of the complex nature of the marine environment.^38^ Polyketidetrichoharzin is the first metabolite reported from a sponge-associated fungus *Trichoderma harzianum* Rifai, isolated from the marine sponge *Mycale cecilia*.^39^ In particular, sponge-associated fungi have yielded novel metabolites with potent anticancer activities.^40^ Gymnastatins A, B and C were the first novel cytotoxic metabolites from a strain of *Gymnasella dankaliensis*, which was isolated from the sponge *Halichondria japonica.*^41^

In the present study, the purification of the compound was carried out by column chromatography. The active fraction was identified, and further purification was performed by HPLC at 17.2 RRT; the purified compound showed a single peak on the chromatogram fixed PDA at 238 nm. 17.2 RRT elution was concentrated under high vacuum, dried and was subjected to spectral analysis, which revealed the mass of the compound to be 418.2. The peak at *m*/*z* = 419 (M + H), the mass spectrum 419.2 [M + H^+^] and 859.5 [2M + Na^+^] atomic mass unit (amu), which corresponds to the exact calculated molar mass of Monacolin X, were observed. Thus peaks were observed at *m*/*z* = 303.20 (M + H-116), 243.1, 199.1 (M + H-220), 143.0 (M + H-275) and 107.1 (M + H-312). Further, the peak at *m*/*z* = 419.2 was further subjected to fragmentation at a collision energy of 20, and peaks were obtained at *m*/*z* = 199.3, 173.2, and 159.2. The isolated compound was dissolved in CDCl_3_ and characterized by ^1^H and ^13^C NMR spectroscopy. The ^1^H-NMR spectrum of the isolated compound suggests the presence of the –COCH_3_ group and one –COCH (CH_3_)CO– group; the presence of these groups can be confirmed by locating a singlet of three protons at 2.25 ppm and a multiplet of one proton at 3.50–3.57 ppm. The ^13^C-NMR spectrum of the isolated compound confirms the carbonyl signal C-3′′ at 204.88 ppm and the corresponding methyne signal C-2′′ at 53.39 ppm. The 4′′-CH_3_ group carbon was observed at 27.33 ppm. The NMR spectroscopic data determined C_24_H_34_O_6_ to be the molecular formula. Further, the UV spectrum of the compound indicates the peak at 238.2 nm, and FTIR analysis revealed all the functional groups present in the compound. Thus, all this data helped us to predict the structure to be 8-(2-(4-hydroxy-6-oxotetrahydro-2*H*-pyran-2-yl)ethyl)-3,7-dimethyl-1,2,3,7,8,8*a*-hexahydronaphthalen-1-yl2-methyl-3-oxobutanoate or monacolin X.^31,32^

In addition, a parametric approach was used to investigate different hallmarks of the proliferation of cellular components affected by apoptosis and cell viability (AO/EB staining and XTT assay), nuclear activity (PI), the activation of cellular organelles (ROS, Δ*ψ*_m_) or the anti-migratory effect. Interestingly, previous reports showed *Monascus* sp. secondary metabolites and monascorubrin, which inhibits cancer promotion in mice;^42^ monascin and ankaflavin showed antiproliferation and proapoptosis effects.^43,44^ It has also been revealed that monacolin K shows a synergistic antitumorigenic activity toward Lewis lung carcinoma cells.^45,46^ In our study, the cytotoxic activity of monacolin X against MCF-7, T47D, MDA-MB-231, MDA-MB-468 and MCF-10A showed a decrease in the number of viable cells due to an increase of cell death and/or decreased cell proliferation, with an IC_50_ lower than 50 μM as compared to non-cancerous MCF-10A cells at 170 μM. This indicated decreased cell proliferation in a dose-dependent manner and the induction of cell death through morphological alterations such as cell shrinkage, membrane blebbing, and rounded and detached cells. Apoptosis is an important physiological process for the maintenance of tissue homeostasis and plays a pivotal role in the pathogenesis of various diseases.^47^ Apoptosis is a programmed cell death, which occurs without eliciting local inflammatory response; in the case of cancer therapy; one of the most important aspects is to induce apoptosis and kill cancer cells *via* apoptosis induction. Hence, apoptosis was confirmed by AO/EB staining, where the IC_50_ value was used to evaluate the apoptosis induced by monacolin X. PI staining was used to differentiate necrotic, apoptotic and normal cells. The characteristics of late apoptosis include some loss of membrane integrity and uptake of PI, binding to the nucleotide pair of guanine and cytosine to stain both DNA and RNA and further fluorescence enhancement.^48^ Monacolin X treatment indicated that more than 40% of cells were dead when compared to the standard control of Dox.

Thus, monacolin X was able to induce nuclear damage and apoptosis, further supporting the role of reactive oxygen species (ROS) and mitochondrial membrane potential (Δ*ψ*_m_). Generally, reactive oxygen species (ROS) have a dual role, either beneficial or harmful depending on their levels of accumulation. ROS generally contributes to cell death either by apoptosis or necrosis at the levels beyond the cellular antioxidant defense mechanisms.^49^ Mitochondria also play a major role in the management of other cellular functions like cell survival and death.^50^ Hence, for checking for the involvement of mitochondria on treatment with monacolin X – induced cell death, the mitochondrial membrane potential (MMP) was studied using Rhodamine-123, where green fluorescence increased in the treatment group representing the potential difference in the mitochondrial membrane. We concluded from our experimental evidence that nuclear damage leading to apoptosis was possibly mediated by ROS, significantly disrupting the mitochondrial membrane.^51^ Cell migration in the scratch assay was carried out to demonstrate the anti-migratory property of monacolin X on MDA-MB-468 and T47D cells, showing an anti-migratory effect when compared to standard. Apart from this, monacolin X demonstrated an excellent antibacterial property in both Gram-positive and negative strains, which was confirmed by the MIC method. This indicates monacolin X having a promising range of biological activity.

## Conclusion

The present study concludes that this is the first study to isolate monacolin X from wild-type fungi associated with marine sponge collected from the coast of the Gulf of Manner, which has a significant anti-cancer property as it is able to induce apoptosis, increased ROS production and mitochondrial membrane changes. Furthermore, monacolin X exhibits excellent anti-migratory property on both high and low metastatic breast cancer cell lines, also showing antibacterial activity on Gram-positive and Gram-negative bacteria. Thus, the present work emphasizes the role of marine sponge-associated fungi secondary metabolites as an important source of leads for anticancer drug discovery.

## Ethical approval

This article does not contain any studies with human participants or animals performed by any of the authors.

## Authors' contributions

Sirpu Natesh Nagabhishek administered the collection of samples, experiential work, and the interpretation of data, while also carrying out the statistical analysis in addition to studying and drafting the manuscript. Dr A. Madankumar designed the study and drafted the manuscript; both authors read and approved the final manuscript.

## Conflicts of interest

The authors declare that no conflicts of interest exist.

## Supplementary Material

RA-009-C8RA09886G-s001
