# Supplementary material for: A novel apoptosis-inducing metabolite isolated from marine sponge symbiont Monascus sp. NMK7 attenuates cell proliferation, migration and ROS stress-mediated apoptosis in breast cancer cells
Source: RSC Adv. 2019 Feb 18;9(11):5878–90. doi: 10.1039/c8ra09886g (PMC9060890; doi:10.1039/c8ra09886g)
Supplement: RA-009-C8RA09886G-s001 [file RA-009-C8RA09886G-s001.pdf]

Electronic Supplementary Material (ESI) for RSC Advances.
This journal is © The Royal Society of Chemistry 2019

Supplementary material

A novel apoptosis inducing metabolite isolated from marine sponge symbiont *Monascus sp.* NMK7 attenuates cell proliferation, migration and ROS stress mediated apoptosis in breast cancer cells

Sirpu Natesh Nagabhishek, Arumugam Madankumar*

Cancer Biology lab, Molecular and Nanomedicine Research unit, Sathyabama Institute of science and technology, Chennai-600119.

<u>Supplementary material</u>

Supplementatry Figure 1

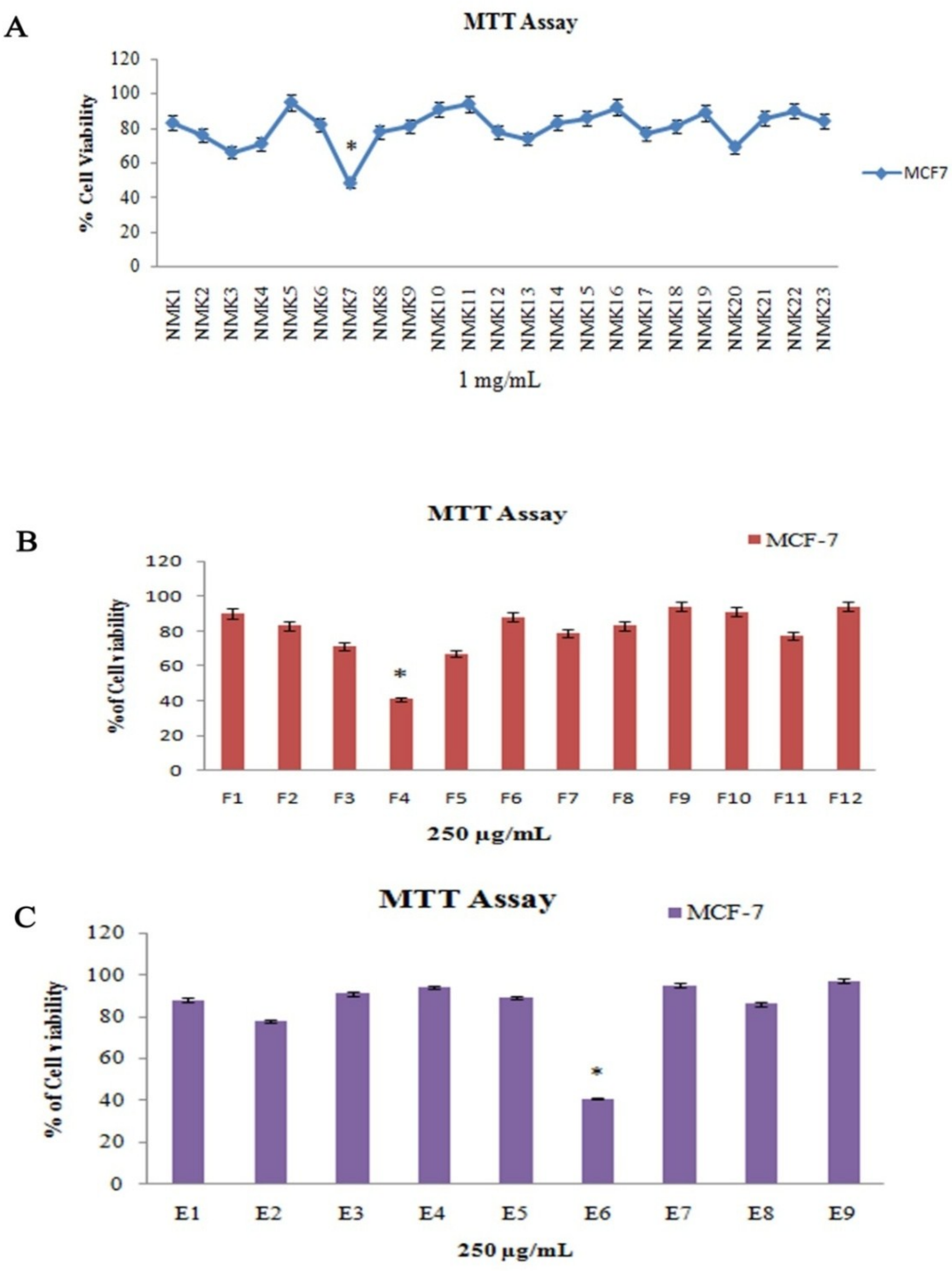

Supplementatry Figure 1 : (A) Shows the MTT assay carried out on the different isolated fungi (NMK1- NMK23) from the marine sponges (Sp1 to Sp 16) out of which the strain showing

potent antiproliferative or cytotoxicity activity was further taken into the study, out of 23 strains NMK7 showed most cytotoxic when treated at 1mg/mL for 24 h on MCF-7 breast cancer cell line. (B) Further NMK7 crude was subjected to column chromatography and eluted out with 12 different fractions (F1 to F12). Entire fraction was assessed for cytotoxicity on MCF-7 cell line with 250 µg/mL for 24 h and found out that F4 has the highest activity. Further fraction 4 (F4) was subjected to HPLC from which 9 elution's (E1 to E9)were eluted out and now each of this was tested for cytotoxcity on MCF-7 cell lines with 250µg/mL for 24 h. we found out that E6 had the highest activity, this E6 elution were collected in large number for the further characterization studies.

Supplementatry Figure 2

A

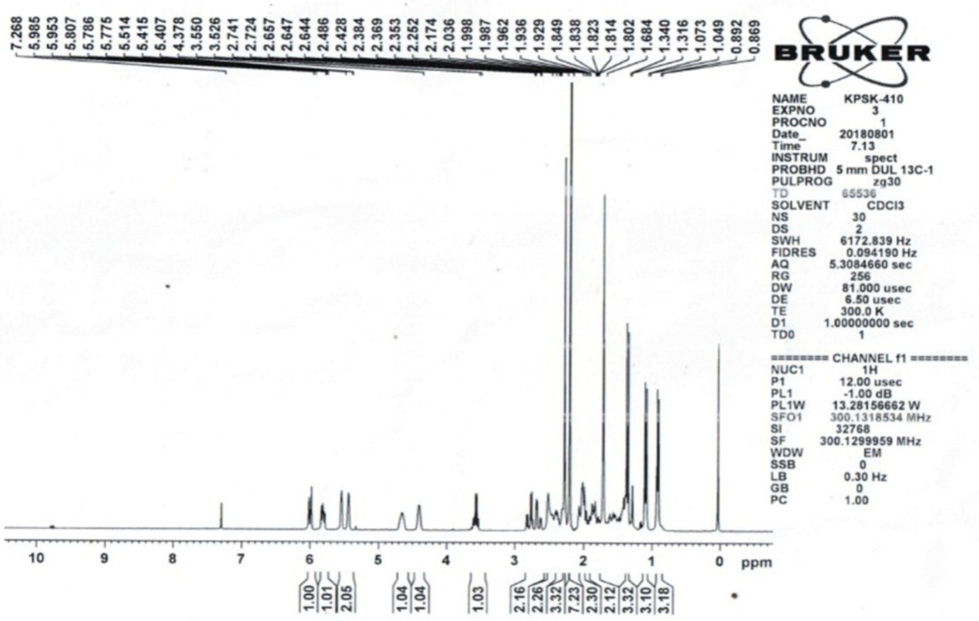

B

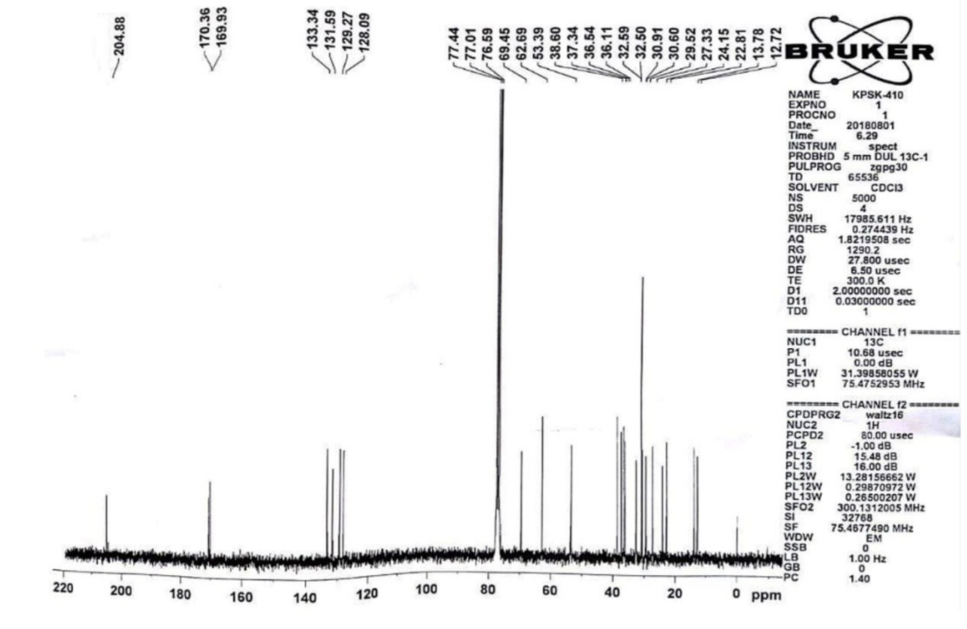

Supplementatry Figure 2: (A) Represents ^{1}H NMR spectrum of NMKD7 (Monacolin X) (300 MHz,CDCL$_3$). (B) ^{13}CNMR spectrum of NMKD7 (monacolin X) (300 MHz,CDCL$_3$).

Supplementatry Figure 3

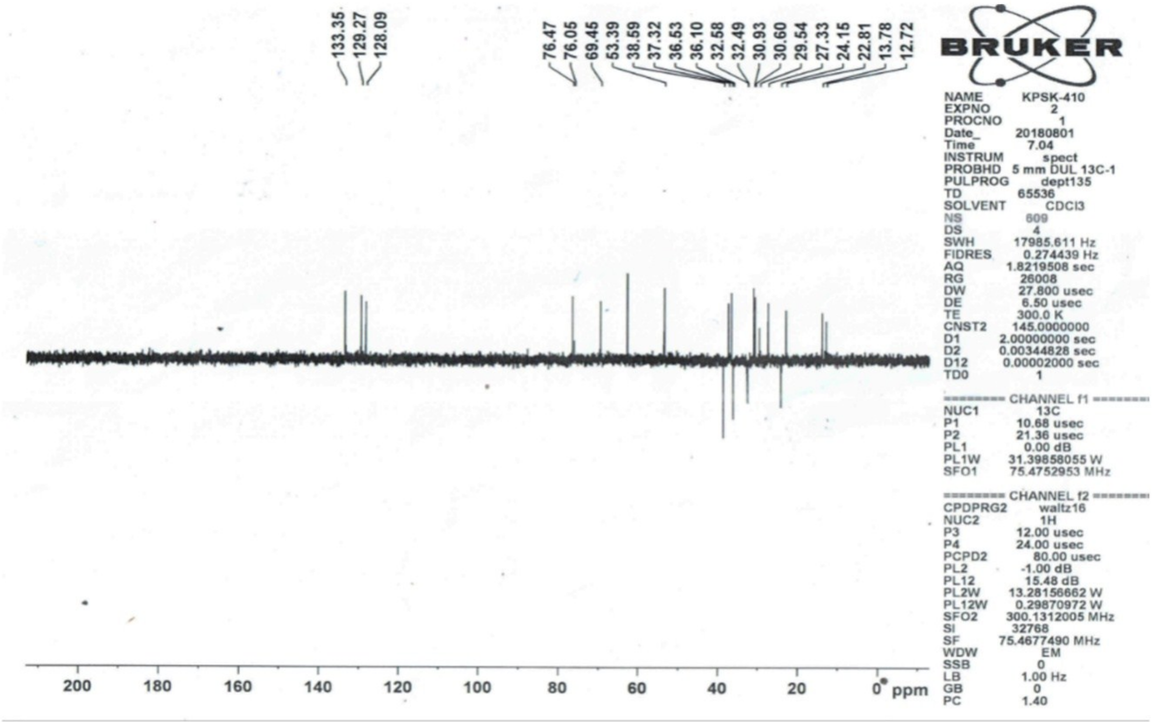

Supplementatry Figure 3: ^{13}C DEPT NMR spectrum of isolated NMKD7 (Monacolin X) (300 MHz,$CDCL_3$)

Supplementatry Figure 4

A

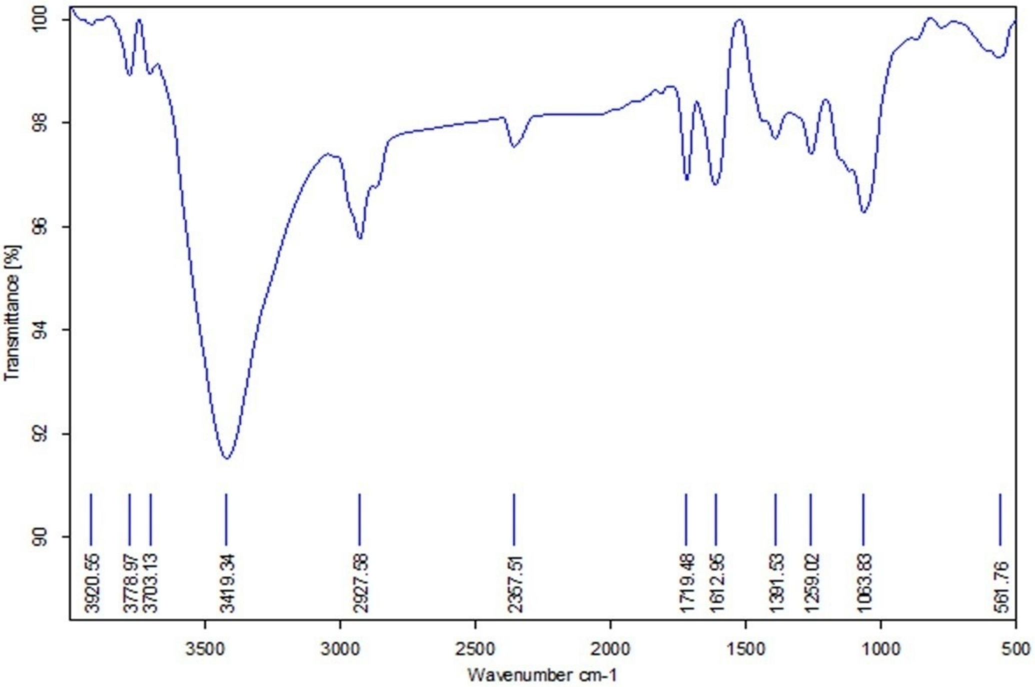

B

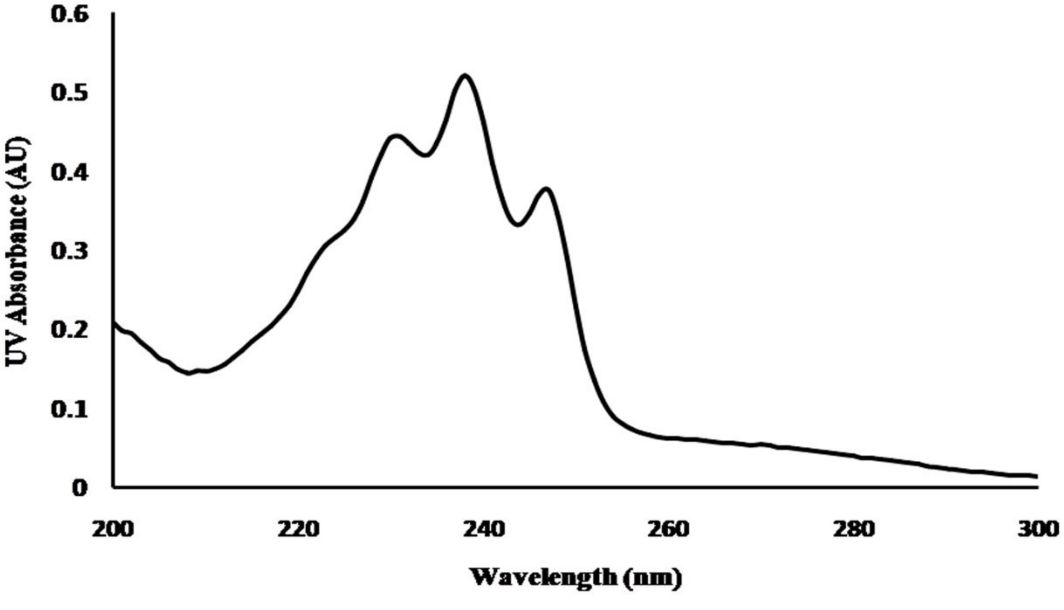

Supplementatry Figure 4 : FT-IR spectrum of NMKD7 (Monacolin X), indicated the presence of The FT-IR spectra of pure compound presented characteristic peak at 3419 cm^{-1} and 3703cm^{-1} (O−H stretch vibration), 2927 cm^{-1} (C−H stretch vibration) and 1259 cm^{-1},1063 cm^{-1} and 1719 cm^{-1} (stretch vibration of −C−O and −C=O carbonyl functional group). 1612 cm^{-1} (C=C) confirming all the functional groups present in the compound. (B) UV spectrum of NMKD7 (Monacolin X). The UV spectrum showed absorption maximum values at 238 nm.
